# Coming together to address neglected tropical diseases

**Published:** 2013

**Authors:** Simon Bush

**Affiliations:** Director of Neglected Tropical Diseases, Sightsavers. Haywards Heath, UK.

**Figure:**
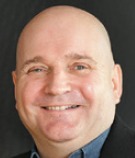
Simon Bush

The NTD NGDO Network^1^ provides a global forum for non-govern mental development organisations (NGDOs) and a wide range of other partners to share information and approaches on the elimination, prevention and control of neglected tropical diseases (NTDs).

The primary mission of the network is to coordinate the activities of members in an attempt to bridge gaps in funding and programme support to endemic countries. The NTD NGDO Network is nota fundraising organisation, as the individual members will continue to raise funds to support specific activities. Rather, it is a convener and facilitator.

The NTD NGDO Network has a number of specific objectives.

- To increase the expansion and effectiveness of advocacy for NTD control by giving NGDOs a unified voice at national and international levels on:
  – comprehensive elimination and control programmes
  – community ownership
  – integrating with development programmes
  – strengthening health systems.
- To facilitate the formation of partnerships among the group's members at the international, regional, and national levels.
- To provide a mechanism for coordination of NGDO activities at national and international levels in order to:
  – avoid duplication of efforts
  – identify opportunities for synergy
  – track progress towards goals
  – identify operational research needs.
- To share technical updates, develop and uphold best practices, and contribute to WHO guidelines to:
  – control and/or eliminate individual NTDs
  – integrate NTD activities
  – promote and support comprehensive NTD control and prevention programmes
  – standardise systems and practices.
- To present, with a unified voice, the common interests and concerns of implementing NGDOs in mechanisms being established for the mobilisation of resources for the implementation of elimination programmes.
- Through its members, to support the development and maintenance of national task forces in NTD endemic countries and assist them to:
  – develop and implement national plans
  – identify gaps and coordinate strategies to meet implementation resource shortfalls.

**Figure:**
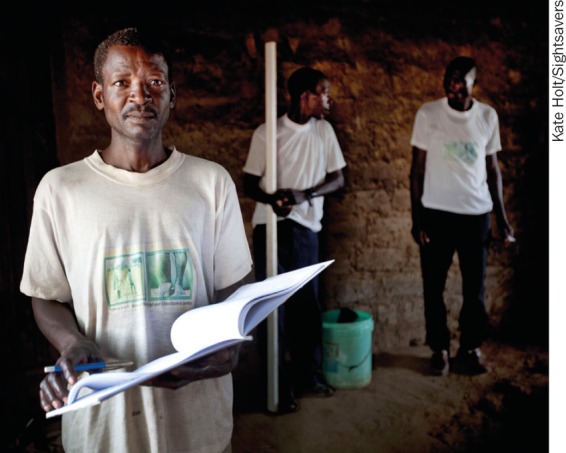
A community directed distributor. NIGERIA

## What does the NGO NGDO Network do?

During the Network's third annual meeting in Sydney, Australia in September 2012^2^, the NTD NGDO Network declared its full and unanimous support for the London Declaration on Neglected Tropical Diseases (The London Declaration).^3^

The members of the NTD NGDO Network have a long history of working with people affected by NTDs and have been among the major innovators in mass drug administration (MDA) for almost 25 years. NGDOs have facilitated the distribution of over 400 million treatments of preventive chemotherapy worldwide in 2011.

NGDOs are critical players in global health, and are uniquely placed, given their field-based programmes and experience, to reach the most under-served – those neglected people that The London Declaration is pledging to reach.

As part of our commitment to the London Declaration, the members of the Network will support the following:

- the finalisation of baseline disease mapping: this is a critical issue to achieve the scale-up of treatment
- the scaling up of mass drug administration coverage: the treatment coverage globally is not reaching the 75% global coverage rate required
- programming solutions to NTDs which will be informed by clinical and operational research
- the building of local capacity which will ensure effective scale-up to achieve elimination targets
- the integration of water, sanitation and hygiene (WASH) programmes into NTD programmes where appropriate. WASH elements are crucial, but often underplayed, elements of the elimination and control of NTDs
- strengthening of health systems, especially at community level
- scale-up of efforts to address the need for surgical intervention, home-based care, and stigma reduction for those suffering from NTDs. NTD programmes need to go beyond MDA
- a commitment to ensuring that neglect is not perpetuated because of someone's gender or disability, whether by denying access to treatment, prevention or morbidity control
- the development of clear guidelines as to where and when to stop treatment. These are essential if elimination targets are to be achieved.

## How to get involved

The next annual meeting will be held in Brighton, UK, 18-20 September 2013, and will include disease-specific meetings on onchocerciasis, trachoma, lymphatic filariasis, schistosomiasis and soil transmitted helminths. The agenda of the meeting will focus on those countries with a high burden of NTDs and the role of NGDOs in achieving the scale-up of treatments. There will be a day to discuss the link between NTDs and sanitation as well as a look at the impact of NTDs on disability and the need to scale-up morbidity control programmes as part of the elimination agenda.

To register for this event go to **http://www.amiando.com/GCLEMDC**

Simon Bush has been the chair of the NTD NGDO Network for the last two years. He hands over to Kim Koporc (Children Without Worms) in September 2013.
